# Misleading Citations and Publication Bias in COVID‐19 in Ophthalmology

**DOI:** 10.1111/jebm.12664

**Published:** 2024-12-15

**Authors:** Giacomo Visioli, Maria Pia Pirraglia, Alessandro Lambiase, Magda Gharbiya

**Affiliations:** ^1^ Department of Sense Organs Sapienza ‐ University of Rome Rome Italy; ^2^ Azienda Ospedaliero‐Universitaria Policlinico Umberto I Rome Italy

1

Misrepresentation of scientific findings can lead to an overestimation of a medical issue, a phenomenon exacerbated when the scientific community is eager for information on a novel pathogen. The COVID‐19 pandemic has led to an unprecedented growth in research output, including numerous studies on potential ocular manifestations [1]. The identification of conjunctivitis as an early symptom of COVID‐19 naturally prompted questions about whether SARS‐CoV‐2 could affect other ocular structures [2]. Initial reports suggesting retinal involvement generated significant interest and debate within the medical community. While such inquiries were legitimate, it is important to approach them with scientific rigor to avoid drawing unwarranted conclusions.

Four years ago, we conducted a study on retinal findings in 46 patients with severe COVID‐19 pneumonia. Our conclusion was unequivocal: we found no retinal alterations attributable to SARS‐CoV‐2 infection [3]. Instead, the observed changes were likely due to systemic conditions such as hypertension or diabetes. Surprisingly, an analysis of the past 2 years' citations revealed that 41.7% misrepresented our article, citing it to claim we supported COVID‐19‐related retinal findings, despite us stating the opposite. These distortions occurred across journals regardless of their prestige, as detailed in Table 1.

**TABLE 1:** Journal metrics and key message alteration in citations of our original article, where we did not identify retinal findings clearly associated with COVID‐19 [3].

Journal metrics	Total (*n* = 36)	Key message alteration (*n* = 15)	No alteration (*n* = 21)	*p* value
H‐Index, median (IQR)	65 (76.8)	68 (84)	62 (84)	0.860
SJR, median (IQR)	0.83 (0.65)	0.72 (0.36)	0.91 (0.68)	0.211
Q1 Journals, *n* (%)	18 (50.0)	5 (33.3)	13 (61.9)	0.176
Ophthalmology field, *n* (%)	19 (52.8)	9 (60.0)	10 (47.6)	0.516

*Note*: Only articles published after January 2022 in English and indexed on Scopus were evaluated.

H‐Index: Hirsch Index; IQR: interquartile range; SJR: SCImago Journal Rank; Q1 Journals: journals ranked in the first quartile (top 25%) based on SJR; Key message alteration: instances where our original article was cited but the main message was misrepresented or attributed statements we did not support.

This case study underscores a critical issue in scientific interpretation: the assumption that simultaneous occurrence indicates causation [4]. Observing retinal abnormalities in patients with COVID‐19 does not necessarily mean that SARS‐CoV‐2 is the etiological or predisposing factor. Especially during a pandemic, when a significant portion of the global population is infected, coincidental occurrences are statistically more likely.

One method to establish a causal relationship is to demonstrate an increased incidence of a condition that correlates specifically with the infection [5, 6]. However, after 4 years of extensive research, no definitive evidence has emerged to support an increased incidence of retinal pathology directly linked to SARS‐CoV‐2 [7]. Findings such as retinal thrombosis or cotton wool spots are more plausibly explained by systemic conditions or comorbidities common in severely ill patients rather than a direct pathogenic role of the virus [8].

Furthermore, it remains unclear whether the ocular findings reported in COVID‐19 patients represent a problem of significant medical relevance. In many cases, these retinal changes are minor, asymptomatic, and do not necessitate specific treatment. Overstating such findings can misdirect scientific focus and may lead to unnecessary alarm among patients. Moreover, there were some highly cited early reports during the pandemic that claimed to identify retinal abnormalities in COVID‐19 patients, but subsequent scrutiny revealed these findings were more likely to represent normal retinal anatomy [9]. These inconsistencies, magnified by widespread citation, have contributed to an exaggerated perception of SARS‐CoV‐2's impact on the retina.

The implications of misinterpreting associations extend beyond ophthalmology. Misleading citations and publication bias can distort the scientific record, misinform clinical guidelines, and possibly impact patient care [10]. To address these issues, it is essential for researchers to rigorously distinguish correlation from causation. Comprehensive studies should control for confounding variables and focus on whether an observed condition occurs at a higher rate in infected individuals compared to the general population, as is often the case when evaluating potential causal effects of vaccines [11, 12]. Further, enhancing tools capable of analyzing citations for relevance and detecting misleading content would be beneficial [13]. Such technologies would assist journals, peer reviewers, and editors in maintaining higher standards of citation accuracy and context. By integrating these solutions into the publication process, we could enhance the integrity of scientific literature [14].

Combining technological advancements with a commitment to publish negative or null results will help provide a balanced and accurate foundation for evidence‐based medicine [15]. By upholding precision in research and publication practices, we can prevent the propagation of misconceptions and ensure that clinical decisions are based on reliable evidence.

## Conflicts of Interest

The authors declare no conflicts of interest.
